# A novel mouse model for an inducible gene modification in the renal thick ascending limb

**DOI:** 10.1152/ajprenal.00250.2022

**Published:** 2023-03-09

**Authors:** Laurent Bourqui, Denise V. Winter, Alex Odermatt, Dominique Loffing-Cueni, Johannes Loffing

**Affiliations:** ^1^Institute of Anatomy, University of Zurich, Zurich, Switzerland; ^2^Division of Molecular and Systems Toxicology, Department of Pharmaceutical Sciences, University of Basel, Basel, Switzerland; ^3^National Centre of Competence in Research “Kidney.CH”, University of Zurich, Zurich, Switzerland

**Keywords:** Ai14 and mT/mG reporter mice, cell lineage tracing, Cre immunostaining, Na^+^-K^+^-2Cl^−^ cotransporter, thin limbs

## Abstract

The thick ascending limb (TAL) is critical for renal control of fluid and ion homeostasis. The function of the TAL depends on the activity of the bumetanide-sensitive Na^+^-K^+^-2Cl^−^ cotransporter (NKCC2), which is highly abundant in the luminal membrane of TAL cells. TAL function is regulated by various hormonal and nonhormonal factors. However, many of the underlying signal transduction pathways remain elusive. Here, we describe and characterize a novel gene-modified mouse model for an inducible and specific Cre/Lox-mediated gene modification in the TAL. In these mice, tamoxifen-dependent Cre (CreERT2) was inserted into the 3′-untranslated region of the Slc12a1 gene, which encodes NKCC2 (Slc12a1-CreERT2). Although this gene modification strategy slightly reduced endogenous NKCC2 expression at the mRNA and protein levels, the lowered NKCC2 abundance was not associated with altered urinary fluid and ion excretion, urinary concentration, and the renal response to loop diuretics. Immunohistochemistry on kidneys from Slc12a1-CreERT2 mice revealed strong Cre expression exclusively in TAL cells but not in any other nephron portion. Cross-breeding of these mice with the mT/mG reporter mouse line showed a very low recombination rate (∼0% in male mice and <3% in female mice) at baseline but complete (∼100%) recombination after repeated tamoxifen administration in male and female mice. The achieved recombination encompassed the entire TAL and also included the macula densa. Thus, the new Slc12a1-CreERT2 mouse line allows inducible and very efficient gene targeting in the TAL and hence promises to be a powerful tool to advance our understanding of the regulation of TAL function.

**NEW & NOTEWORTHY** The renal thick ascending limb (TAL) is critical for renal control of fluid and ion homeostasis. However, the underlying molecular mechanisms that regulate TAL function are incompletely understood. This study describes a novel transgenic mouse model (Slc12a1-creERT2) for inducible and highly efficient gene targeting in the TAL that promises to ease physiological studies on the functional role of candidate regulatory genes.

## Graphical abstract

**Figure:**
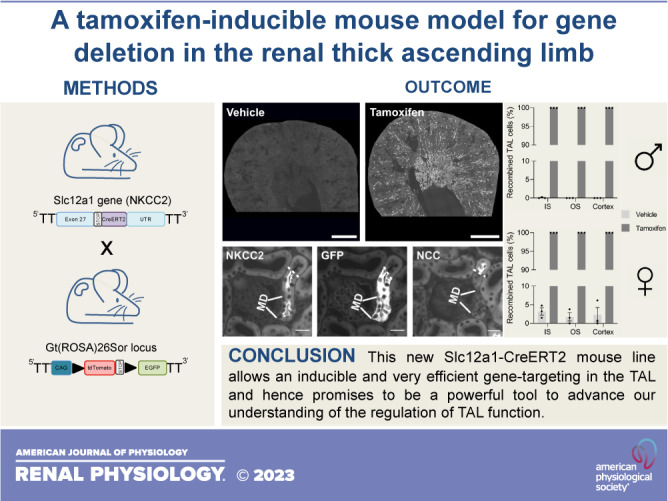

## INTRODUCTION

The thick ascending limb (TAL) plays a pivotal role for the maintenance of ion and water homeostasis (1). The TAL reabsorbs large quantities of the Na^+^, Ca^2+^, and Mg^2+^ that have been filtered at the glomerulus. Although the TAL is water impermeable, TAL function is also critical for renal water reabsorption. The high Na^+^ transport rate of the TAL contributes significantly to the generation of the high osmotic gradient in the renal medulla, which finally drives water transport in the collecting duct and hence urinary concentration (1). Furthermore, the TAL forms the macula densa, which is critical for tubuloglomerular feedback (2). Na^+^ transport along the TAL depends on the activity of the bumetanide-sensitive Na^+^-K^+^-2Cl^−^ cotransporter (NKCC2) in the apical plasma membrane (3). Na^+^ transport via NKCC2 is driven by Na^+^-K^+^-ATPase localized in the basolateral plasma membrane and requires that the cotransported K^+^ and Cl^−^ can exit the cell via the apical renal outer medullary K^+^ (ROMK) channel and basolateral Cl^−^ channel CLC-Kb, respectively (1). Although the activity of NKCC2 is electroneutral, the apical K^+^ and basolateral Cl^−^ release via ROMK and ClC-Kb generates a lumen-positive transepithelial electrochemical gradient that drives the paracellular reabsorption of Ca^2+^ and Mg^2+^ and, to some extent, also of Na^+^ (1). The relevance of the TAL for ion and water homeostasis is evidenced by Bartter’s syndrome, a human hereditary renal tubulopathy in which loss-of-function mutations in NKCC2, ROMK, ClC-Kb, or other proteins relevant for TAL function cause life-threatening renal salt and fluid wasting combined with hypercalciuria and hypokalemic metabolic alkalosis (3).

The function of the TAL is regulated by several hormones including glucocorticoids, vasopressin, catecholamines, atrial natriuretic peptide, glucagon, and parathormone (4). Likewise, several nonhormonal factors like extracellular and intracellular ion concentrations, acid-base status, and proteins such as uromodulin impact TAL function (1, 5). These hormonal and nonhormonal factors mediate their effects via complex signal transduction pathways that include several kinases like protein kinase A, protein kinase C, Ste20- and SPS1-related proline and alanine-rich kinase, and oxidative stress-responsive kinase as well as phosphatases like protein phosphatase 1 and protein phosphatase 3 (calcineurin) (6–8). The functional role and interplay of these various signaling pathways are yet not fully understood. Moreover, recent transcriptomic and proteomic analysis of isolated single cells and microdissected tubules has identified numerous additional candidate genes possibly involved in the regulation of TAL function (9, 10).

Targeted gene modification in mice is a critical tool for analysis of the functional significance of particular candidate genes in vivo. The Cre-LoxP system is one of the most widely used systems that allows both spatial (i.e. cell type specific) and temporal control of gene expression in an organism (11). The system takes advantage of transgenic expression of a Cre recombinase that specifically targets genes that have been flanked by two artificial LoxP sites. In this system, spatial control is achieved by driving Cre expression by a cell type-specific promoter. Temporal control can be achieved by fusing of Cre to a modified estrogen receptor (ER) that is insensitive to natural estrogens but activated after application of synthetic steroids such as tamoxifen (CreERT) (12). In vivo, applied tamoxifen is converted into the active metabolite 4-hydroxy-tamoxifen (4-OHT) that binds to the CreERT fusion protein, which translocates it from the cytoplasm to the nucleus, where Cre can then target genes flanked by LoxP sites (11). ERT2 is a further improved version of ERT, in which two additional mutations confer a 10-fold increase in the sensitivity of the receptor to 4-hydroxy-tamoxifen (13). This ensures high recombination rates already at low-dose tamoxifen treatment and hence helps to avoid side effects of tamoxifen.

For kidney research, several Cre mouse lines have been engineered, which target specific nephron segments such as the proximal tubules (14), distal convoluted (DCT) tubule (15, 16), or collecting system (17). However, the scientific community lacks a mouse line for specific and conditional targeting of the TAL. Two mouse lines expressing Cre recombinase under the control of the uromodulin or Tamm-Horsfall protein promoter have been developed (18, 19). However, uromodulin is expressed in the TAL and DCT (20), and hence these mice do not allow TAL-specific targeting of gene products. Lu et al. (21) have developed a mouse line in which Cre is constitutively expressed under the TAL-specific NKCC2 promoter, which ensures good spatial but no temporal control of gene modification. In the present study, we characterized a novel mouse line in which CreERT2 was inserted into the 3′-untranslated region (3′-UTR) for the Slc12a1 gene, which permits inducible and highly efficient gene modification specifically in the TAL.

## MATERIALS AND METHODS

### Animals

**Figure 1.**
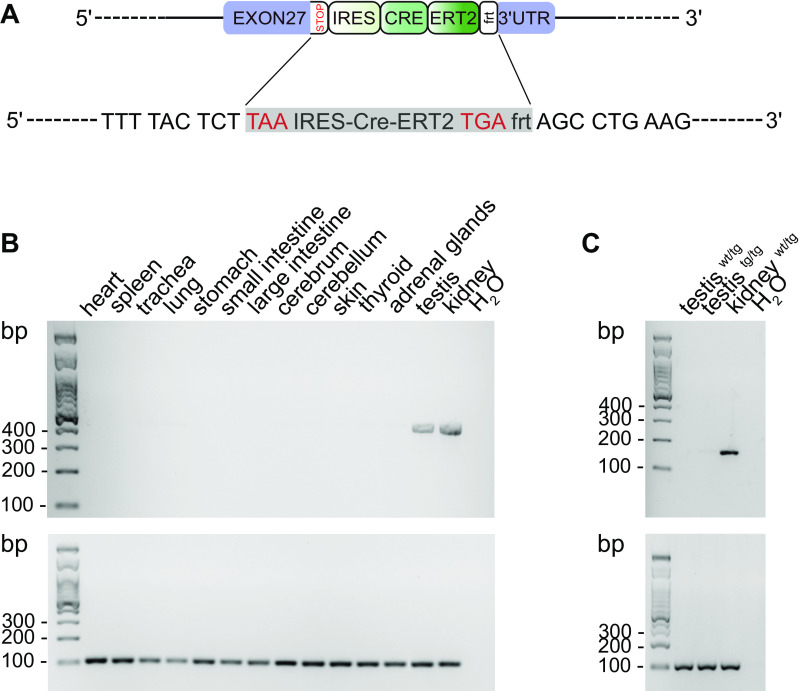
Cre expression in the Slc12a1-CreERT2 mouse model. *A*: schematic representation of the gene modification. The CreERT2 sequence with its own internal ribosome entry site (IRES) was inserted directly after the stop codon of the 27th exon of the Slc12a1 gene. *B*: Cre expression in multiple organs from Slc12a1-CreERT2^wt/tg^ male mice (*n* = 5) as assessed by agarose gel electrophoresis after amplification of Cre (*top*) and GAPDH (*bottom*) from total tissue RNA and reverse transcribed into cDNA. *C*: Na^+^-K^+^-2Cl^−^ cotransporter (NKCC2) expression in the kidney of a Slc12a1-CreERT2 heterozygous (wt/tg) male mouse (*n* = 1) but not in the testis of Slc12a1-CreERT2 heterozygous (wt/tg) and homozygous (tg/tg) male mice (*n* = 1 each). NKCC2 RT-PCR and agarose gel electrophoresis from total kidney and testis RNA are shown. tg, transgenic; wt, wild-type.

All mice were kept in the animal husbandry facility of the Laboratory Animal Service Center of the University of Zurich with free access to water and food ad libitum. The B6-Slc12a1 tm(cre/ERT2)Jlo (Slc12a1-CreERT2) mouse line was custom made by Ozgene (Perth, WA, Australia) and bred in a C57Bl/6 background. In these mice, the Cre-ERT2 sequence was inserted by homologous recombination into the 3′-UTR of the 27th exon of the SLC12a1 gene (Fig. 1*A*). The tdTomato (22) and mT/mG (23) reporter mouse lines were obtained from the Jackson Laboratories Repository (Bar Harbor, ME). For experimental breedings, wild-type (wt) mice (Slc12a1-creERT2^wt/wt^) were mated with transgenic (tg) mice (wt/tg) (Slc12a1-creERT2^wt/tg^). The offspring of these litters was then pooled into experimental groups that were matched for age and sex. Animal experiments were conducted according to Swiss law and were approved by the veterinary administration of the Canton of Zurich (Kantonales Veterinäramt), Switzerland.

### Genotyping

Biopsies were taken from the toes and digested in 200 mM Tris·HCl, 100 mM EDTA, 0.5% Tween 20, and 1% proteinase K and heated to 55°C overnight. Extracts of genomic DNA were amplified by PCR with the appropriate primers (Supplemental Table S1).

### Tamoxifen Induction

At the age of 6 wk, mice received by gastric gavage 50 µL of either 40 mg/mL/day tamoxifen (T5648, Sigma-Aldrich, St. Louis, MO) diluted in 10% ethanol and 90% sunflower oil (induction group) or 100% sunflower oil only (control group) for five consecutive days.

### Metabolic Data and Water Restriction Test

Seven-week-old mice were individually kept in metabolic cages (Tecniplast S.p.A., Buguggiate, Italy) with free access to tap water and wet food [standard laboratory chow mixed in water (1:1), Ssniff, Spezialddiäten]. After 2 days of acclimatization, 24-h urine and feces were collected, and body weight, food, and water consumption were assessed. After 2 days, some mice were challenged by mild water restriction. For this, the water bottle was removed for 24 h but the mice were maintained with free access to the wet food. In contrast to complete water deprivation, this type of a controlled water limitation is well tolerated by rodents and ensures the assessment of urinary concentrating capacity without a confounding activation of sympathetic or other humoral stress responses (24).

### Bumetanide Response Test

Seven-week-old male mice were studied. On *day 1*, all mice received a single intraperitoneal injection (10 µL/g body wt) of the vehicle solution [mixture of polyethylenglycol 300 and NaCl (0.9%) at a 1:2.5 ratio] with subsequent urine sampling in metabolic cages for 4 h. On *day 2*, all mice were injected with bumetanide at 40 μg/g body wt (diluted in vehicle) with subsequent urine collection for 4 h.

### Organ Harvesting

Mice were anesthetized with isoflurane (2%–4%, 0.5 L/min, Provet). Moreover, mice received a subcutaneous injection of buprenorphine (Temgesic, 0.05–0.4 mg/kg body wt, Indivior) for additional analgesia. For subsequent biochemical analysis, animals were perfused via the left ventricle with PBS before the kidneys and other organs were harvested and flash frozen in liquid nitrogen. For subsequent immunohistochemistry, the kidneys and other organs were fixed by retrograde prefusion through the abdominal aorta using 3% paraformaldehyde in 0.1 M phosphate buffer (0.2 M NaH_2_PO_4_·H_2_O, 0.2 M Na_2_HPO_4_·H_2_O, and 0.1 M CaCl_2_, pH 7.4, 300 mosmol/kgH_2_O). Five minutes after perfusion was begun, the fixative was rinsed out by 0.1 M phosphate buffer.

### Blood and Urine Analysis

Blood was collected from the inferior vena cava using heparinized syringes. Blood gas and ion concentrations were determined using the AL825Flex Blood Gas Analyzer (Radiometer, Brønshøj, Denmark). The remaining blood was centrifuged for 10 min at 3,380 *g*. Afterward, the plasma was transferred into Eppendorf tubes and frozen at −80°C until further use. Urine Na^+^, K^+^, and Ca^2+^ were measured with flame photometry using EFOX 5053 (Eppendorf, Hamburg, Germany). Urine creatinine was assessed by the Jaffe method. Urine and plasma Mg^2+^ were measured at the Zurich Centre for Integrative Rodent Physiology with a SYNCHRON LX System(s) (Beckman Coulter Ireland, Galway, Ireland). Urine aldosterone was measured using an ELISA kit (Cayman Chemical, Ann Arbor, MI). Plasma steroid levels were determined by liquid chromatography-tandem mass spectrometry (UPLC-MS/MS), as previously described (25). Urine parameters were normalized to body weight and urine volume.

### Tissue Homogenization

Frozen organs were transferred into ice-cold tubes filled with ceramic beads (32 Magna Lyser Green Beads, Roche, Mannheim, Germany) and lysis buffer. For proteins (kidney), we used detergent-free lysis buffer containing 200 mM mannitol, 80 mM HEPES, and 40 mM KOH supplemented with protease inhibitor (Complete Ultra, Roche) and phosphatase inhibitor (PhosSTOP, Roche). For RNA extraction (kidney or organ), we use the lysis buffer provided by the kit. Samples were homogenized using a Precellys 24 tissue homogenizer (Bertin Instruments, Montigny-le-Bretonneux, France).

### RNA Extraction and Quantitative PCR

Total RNA was isolated from homogenized organs using either the SV Total RNA Isolation System (Z3100, Promega, Madison, WI) or Nucleospin RNA isolation (Macherey Nagel, Düren, Germany) according to the manufacturer’s protocol. With exception for the kidney, isolated RNA was treated once more with DNAse and further purified using an RNA purification kit (Macherey Nagel, Düren, Germany) according to the manufacturer’s protocol. Equal concentrations of isolated RNA (500 ng) were reverse transcribed into cDNA using the high-capacity cDNA Reverse Transcription Kit (Thermo Fisher, Vilnius, Lithuania) according to the manufacturer’s protocol. cDNA was then 1:5 diluted for further utilization. Threshold cycle (*C_T_*) values of the genes of interest and of a housekeeping gene (GAPDH or TBP) were assessed by real-time quantitative PCR using the LightCycler 480 (Roche). Primers are provided in Supplemental Table S1.

### Generation of a New Anti-Cre Antibody

**Figure 2.**
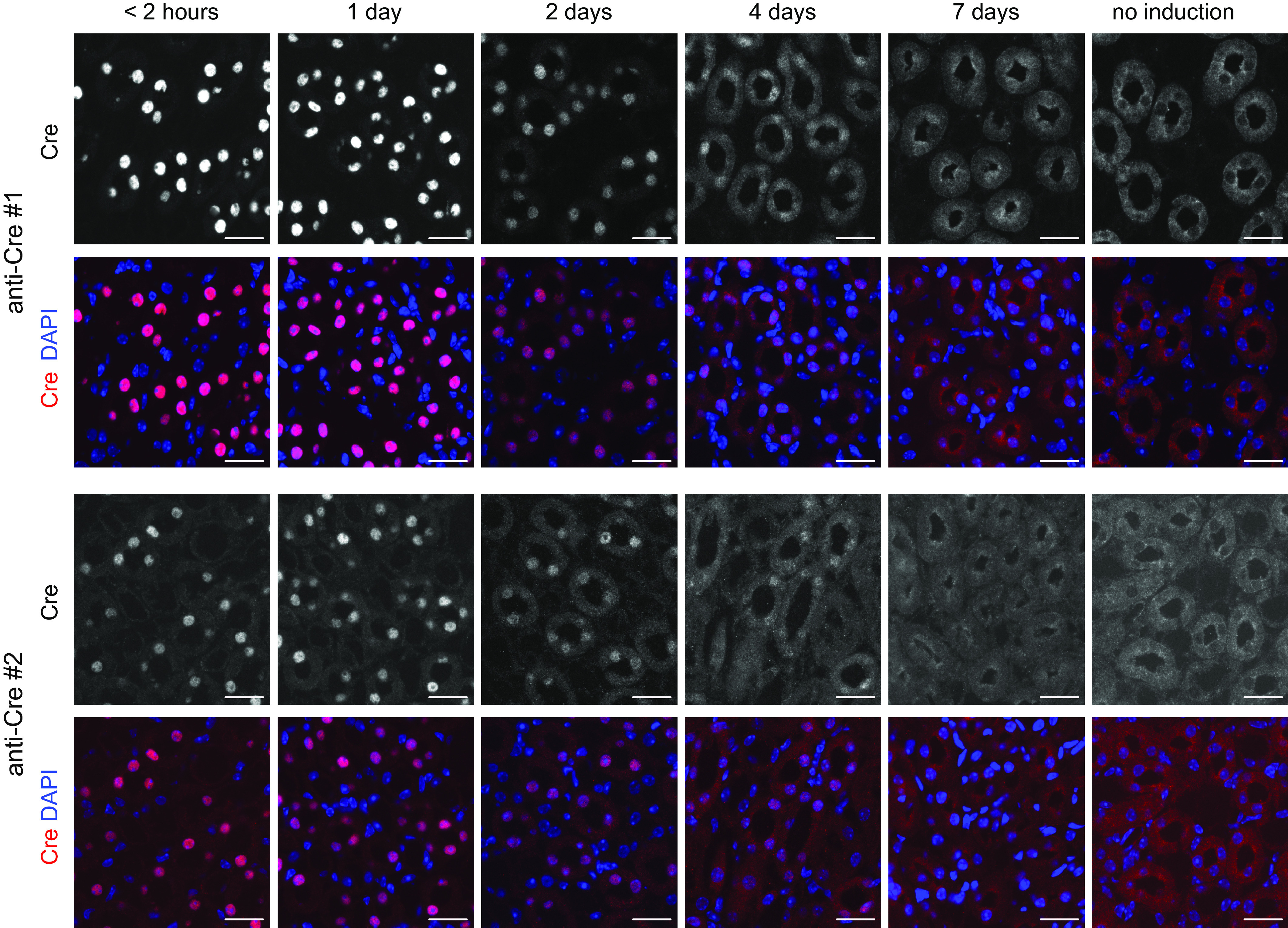
Immunofluorescent detection of Cre in cryosections of kidneys from male Slc12a1-CreERT2^wt/tg^ mice at different time points after 5 daily tamoxifen gavages and in a female Slc12a1-CreERT2^wt/tg^ mouse without tamoxifen induction. All images show cross sections of thick ascending limbs in the inner stripe of the renal outer medulla and are representative for two mice (one female and one male) that were analyzed. The two *top* images show the previously characterized (26) reference Cre antibody (anti-Cre No. 1), whereas the two *bottom* images show the new Cre antibody (anti-Cre No. 2) described in this study. Time points indicate the time period elapsed after the last tamoxifen gavage. Images were captured with a ×20 objective. Scale bars = 25 µm.

As the rabbit anti-Cre antibody generously provided by Dr. G. Schütz and Dr. S. Berger (26) runs short, a new rabbit anti-Cre antibody was custom made by Pineda Antikörper-Service (Berlin, Germany). The antibody was raised against a peptide (EVRKLNMDMFRDRQAFSEHC) corresponding to amino acids 22–40 of Cre to which a cysteine-residue was added at the COOH terminus to allow coupling of the peptide to keyhole limpet hemocyanin for immunization. The new Cre antibody showed a similar staining pattern as the previously generated reference antibody (Fig. 2).

### Western Blot Analysis

Protein concentrations were measured with the Bradford protein assay (Interchim, Montluçon, France) and equilibrated with Coomassie staining (National Diagnostics, Atlanta, GA). Proteins (50 µg) were denatured in Laemmli buffer [10% SDS, 0.5 M Tris·HCl (pH 6.8), 0.5% bromophenolblue, and β-mercaptoethanol]. For detection of the epithelial Na^+^ channel (ENaC), proteins were boiled at 90°C for 5 min. Proteins were separated by electrophoresis using 8%–12% acrylamide gel and transferred to nitrocellulose membrane (Bio-Rad, Hercules, CA). After protein transfer, membranes were stained with REVERT Total Protein Stains (LI-COR Biosciences, Lincoln, NE), imaged with the Odyssey IR imaging system (LI-COR Biosciences), blocked for 20–45 min with Odyssey Blocking solution [PBS diluted (1:1) at room temperature], and incubated with the primary antibodies (Table 1) diluted in Odyssey Blocking solution [PBS (1:1) supplemented with Tween 20 (1:1,000) at 4°C] overnight. After repeated washes with PBS, the membrane was incubated for 1 h at room temperature with the secondary antibodies (Table 1) diluted in casein-blocking solution (Sigma-Aldrich), which had been prediluted 1:10 in distilled water. After repeated washes in PBS, immunoreactive bands were imaged. Signal densities were measured using Fiji ImageJ (v1.53c, Bethesda, MD) and normalized against REVERT protein staining.

**Table 1.** List of antibodies

Antibody	Host	Dilution for Western Blot	Dilution for Immunohistochemistry	Source
Anti-total NCC	Rabbit	1:5,000	1:5,000	J. Loffing (27)
Anti-pT58-NCC	Rabbit	1:5,000		J. Loffing (27)
Anti-total NKCC2	Rabbit	1:5,000	1:5,000	J. Loffing (28)
Anti-pT101-NKCC2	Rabbit	1:1,500		J. Loffing (29)
Anti-TRPM6	Rabbit	1:2,000		J. Loffing (30)
Anti-α-ENaC	Rabbit	1:5,000		J. Loffing (27)
Anti-β-ENaC	Rabbit	1:20,000		J. Loffing (28)
Anti-γ-ENaC	Rabbit	1:20,000		J. Loffing (28)
Anti-ROMK	Rabbit	1:1,000		J. Loffing (31)
Anti-AQP2	Rabbit	1:20,000		J. Loffing (28)
Anti-Cre (No. 1)	Rabbit		1:5,000	G. Schütz (26)
Anti-Cre (No. 2)	Rabbit		1:10,000	This study
Cy3-conjugated-anti-rabbit	Goat		1:1,000	Jackson ImmunoResearch Laboratories (No. 111-165-144)
Cy5-conjugated-anti-rabbit	Goat		1:1,000	Jackson ImmunoResearch Laboratories (No. 111-175-144)
IRDye800-conjugated anti-rabbit	Goat	1:10,000		LI-COR (No. 926-32211)

AQP2, aquaporin; ENaC, epithelial Na^+^ channel; NCC, Na^+^-Cl^−^ cotransporter; NKCC2, Na^+^-K^+^-2Cl^−^ cotransporter; ROMK, renal outer medullary K^+^ channel; TRPM6, transient receptor potential cation channel subfamily M member 6.

### Immunohistochemistry

Perfusion-fixed kidneys were sectioned in slices (0.5 mm thick), mounted on cork plates, flash frozen at −80°C in liquid propane, and kept at −80°C. Sections (4 μm thick) were cut from the frozen kidneys using a cryostat (Microm HM 550, Thermo Fisher, Walldorf, Germany) and mounted on Superfrost slides (Epredia, Braunschweig, Germany). Sections were blocked with normal goat serum (1:10 diluted in 2% BSA in PBS) for 10–30 min and incubated overnight with the primary antibodies (Table 1) diluted in 1% BSA in PBS in a humid chamber at 4°C. After repeated washes in PBS, sections were incubated with fluorescent dye-conjugated secondary antibody diluted in 1% BSA in PBS supplemented with DAPI (1:1,000) in a humid dark chamber for 2–3 h at room temperature. After final washes in PBS, coverslips were mounted using DAKO Glycergel Mounting Medium (Agilent, Santa Clara, CA). Images were captured using a Leica DM6000 B fluorescence microscope on a Leica DFC350 FX fluorescence monochrome digital camera (Leica Microsystems, Wetzlar, Germany). Image processing was done with Fiji ImageJ (v1.53c).

### Quantification of the Recombination Rate

Fixed kidneys from mT/mG × Slc12a1-CreERT2 mice that had been treated with either vehicle (*n* = 3 females and *n* = 3 males) or tamoxifen (*n* = 3 females and *n* = 3 males) were analyzed 2 wk after the last gavage. In cryosections from each mouse, 100 TALs were randomly selected and the number of nucleated cells with recombination versus the number of all nucleated cells per TAL was determined and expressed in percent.

### Statistics

Slc12a1-CreERT2^wt/wt^ and Slc12a1-CreERT2^tg/tg^ mice were compared using a Student *t* test unless otherwise indicated (GraphPad Prism, v.9.2.0, La Jolla, CA). Data are expressed as means ± SE. Statistical tests with a *P* value of <0.05 were considered significant.

## RESULTS

### Strong Cre Transcription in Kidneys of Slc12a1-Cre-ERT2 Mice

First, we assessed, by RT-PCR, expression of Cre recombinase in various organs of noninduced *Slc12a1-Cre-ERT2* heterozygous (wt/tg) mice (*n* = 5 male mice/group). As expected, Cre was highly abundant in the kidneys (Fig. 1*B*). Surprisingly, a faint band was also observed in the testis. Interestingly, this Cre expression was independent from NKCC2, the expression of which was strictly restricted to the kidney (Fig. 1*C*). Transcription of Cre in the testes of Slc12a1-Cre-ERT2 mice coincided with trends for decreased plasma levels of testosterone and androstenedione (Table 2). Nevertheless, male Slc12a1-Cre-ERT2 mice remained fertile and produced offspring when used for breeding.

**Table 2.** Body weight, food/water intake, and plasma and urine parameters

	wt (*n* = 11)	wt/tg (*n* = 13)	*P* Value
pH	7.36 ± 0.02	7.35 ± 0.01	0.7905
Pco_2_, kPa	4.91 ± 0.27	4.99 ± 0.15	0.8773
Po_2_, kPa	5.45 ± 0.19	6.22 ± 0.23	0.1628
cBase, mmol/L	-4.97 ± 0.33	-4.59 ± 0.43	0.7918
Anion gap. mmol/L	22.11 ± 0.33	22.11 ± 0.49	0.6999
Plasma aldosterone, nM	0.23 ± 0.04	0.23 ± 0.05	0.9714
Plasma testosterone, nM	9.70 ± 1.88	2.79 ± 0.69	0.0882
Plasma androstenedione, nM	0.43 ± 0.08	0.13 ± 0.03	0.0791
Plasma progesterone, nM	2.24 ± 0.22	1.98 ± 0.16	0.6145
Plasma Na^+^, mmol/L	149.45 ± 0.15	149.23 ± 0.21	0.6299
Plasma K^+^, mmol/L	4.08 ± 0.06	3.94 ± 0.05	0.3552
Plasma Ca^2+^, mmol/L	1.31 ± 0.01	1.33 ± 0.01	0.4569
Plasma Cl^−^, mmol/L	107.82 ± 0.43	107.31 ± 0.43	0.6469
Plasma Mg^2+^, mmol/L	2.11 ± 0.02	2.05 ± 0.02	0.2695

	wt (*n* = 6)	wt/tg (*n* = 9)	*P* Value
Body weight, g	27.6 ± 1.50	26.8 ± 1.3	0.3142
Water intake, mL·g body wt^−1^	0.21 ± 0.01	0.20 ± 0.01	0.5472
Food intake normalized to body weight	0.16 ± 0.00	0.16 ± 0.01	0.5845
Urine volume, mL·g body wt^−1^	0.08 ± 0.01	0.09 ± 0.00	0.4229
Urine osmolality, mosmol/kgH_2_O	1956 ± 103	1661 ± 48	0.2341
Urine aldosterone, ng·g body wt^−1^	44.91 ± 3.22	59.55 ± 4.21	0.1449
Urine creatinine, μmol·g body wt^−1^	0.17 ± 0.01	0.19 ± 0.01	0.4820
Urine Na^+^, μmol·g body wt^−1^	9.17 ± 0.28	8.9 ± 0.47	0.7811
Urine K^+^, μmol·g body wt^−1^	20.49 ± 0.73	21.71 ± 0.73	0.5734
Urine Ca^2+^, μmol·g body wt^−1^	0.12 ± 0.01	0.10 ± 0.01	0.2347
Urine Mg^2+^, μmol·g body wt^−1^	1.54 ± 0.12	1.87 ± 0.11	0.3053

Data are from male mice and are expressed as means ± SE. Food and water intake and creatinine were normalized to body weight. Urine ions and creatinine were normalized to body weight and to urine volume. Urine was collected over 24 h. Data were compared using a Student’s *t* test. tg, transgenic; wt, wild-type.

### Strong Cre Recombinase Expression in TALs of Slc12a1-Cre-ERT2 Mice

**Figure 3.**
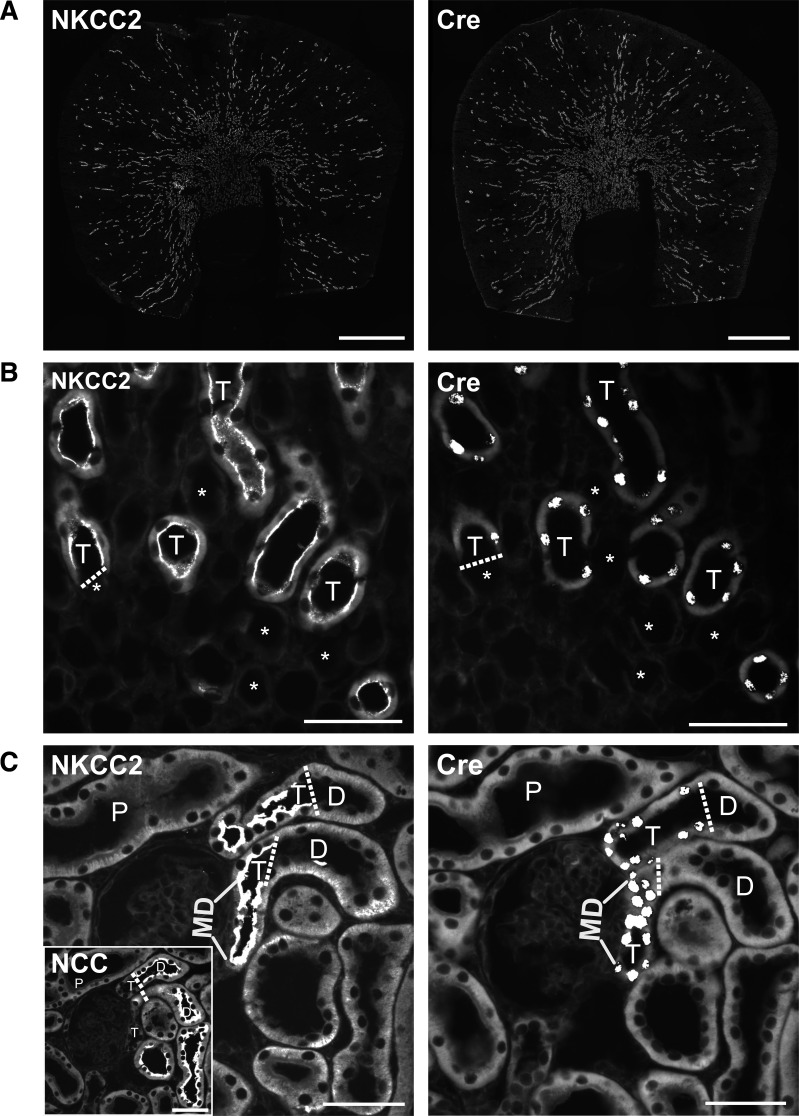
Immunofluorescent detection of Cre in cryosections of the kidney of a Slc12a1-CreERT2^wt/tg^ male mouse immediately after 5 days of repeated tamoxifen gavage. *A*: overviews on consecutive cryosections showing the same distribution patterns for Na^+^-K^+^-2Cl^−^ cotransporter (NKCC2) and Cre (anti-Cre No. 1). *B*: high magnification of the consecutive cryosections revealed that the nuclear Cre expression started precisely at the transition (dashed line) from the NKCC2-negative thin ascending limb (*) to the NKCC2-positive thick ascending limb (T). *C*: high magnification of the consecutive cryosections showed that nuclear Cre localization ceased at the transition (dashed line) from the NKCC2-positive thick ascending limb (T) to the NKCC2-negative but Na^+^-Cl^−^ cotransporter (NCC)-positive (*inset*) distal convoluted tubule (D). The macula densa (MD) cells were also Cre positive. P, proximal tubules. Images were captured with a ×20 objective in *A* and *C* and with a ×40 objective in *B*. Scale bars = 1,000 μm in *A* and 50 μm in *B* and *C*. Images are representative for 3 Slc12a1-CreERT2^wt/tg^ male mice that had been analyzed.

Cre recombinase expression was assessed by immunohistochemistry in the kidneys of heterozygous Slc12a1-Cre-ERT2 mice immediately after the last tamoxifen gavage. Three consecutive kidney cryosections were probed with antibodies against Cre recombinase or total NKCC2 or total Na^+^-Cl^−^ cotransporter (NCC; Fig. 3). Immunostaining for Cre recombinase was mainly seen in the renal cortex and outer medulla and showed a similar distribution pattern as the signal seen with NKCC2 immunostaining (Fig. 3*A*). Higher magnifications revealed that the signal of Cre recombinase was limited to the nuclei of NKCC2-positive cells (Fig. 3, *B* and *C*). Cre expression started precisely at the abrupt transition from the very flat epithelium of the thin ascending limb to the taller epithelium of the TAL (Fig. 3*B*) and ended at the sharp transition from the NKCC2-positive TAL to the NCC-positive DCT. Cells of the macula densa were also Cre positive (Fig. 3*C*). For the design of future experiments, we also analyzed how rapid Cre recombinase redistributed back from the cell nucleus to the cytoplasm after the stop of tamoxifen application. We used two different Cre antibodies, which both revealed that nuclear Cre localization remained rather strong during the first 2 days after the last tamoxifen administration but that nuclear Cre localization progressively disappeared toward *day 7* after the stop of tamoxifen application (Fig. 2).

**Figure 4.**
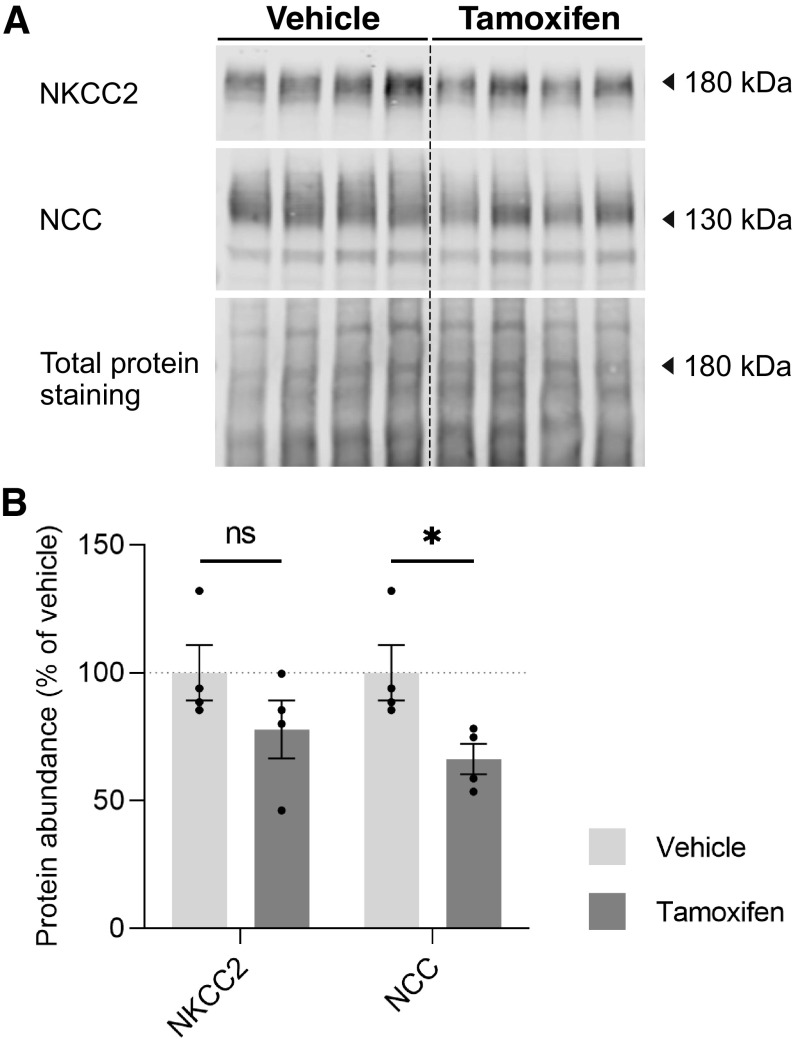
Na^+^-K^+^-2Cl^−^ cotransporter (NKCC2) and Na^+^-Cl^−^ cotransporter (NCC) protein abundance in female Slc12a1-CreERT2^wt/wt^ mice after 5 daily doses of vehicle or tamoxifen. *A*: immunoblots for total NKCC2 and NCC in total kidney homogenates of vehicle- and tamoxifen-treated mice. *B*: densitometry analysis of the immunoblots. Band signals were normalized to total protein staining with REVERT solution (see materials and methods) and expressed as a percentage of the values from vehicle-treated mice. Data are expressed as means ± SE and were compared using a Student’s *t* test; *n* = 4 per group. *P < 0.05. ns, not significant.

Tamoxifen is a potent ER blocker. As estrogens have been shown to modulate NCC and NKCC2 protein abundance in the kidney and as female mice and rats appear to express more NKCC2 and NCC than males (32, 33), we also tested for the impact of our tamoxifen application protocol on the protein abundance of these two solute transporters. Six-week-old female wild-type mice received daily gavages of either vehicle or tamoxifen before the kidneys were removed immediately after the fifth and last gavage. Tamoxifen slightly reduced NKCC2 and NCC expression, but only for NCC did the difference between groups reach statistical significance (Fig. 4).

### High Cre Recombinase Activity in the TAL of Slc12a1-Cre-ERT2 Mice

**Figure 5.**
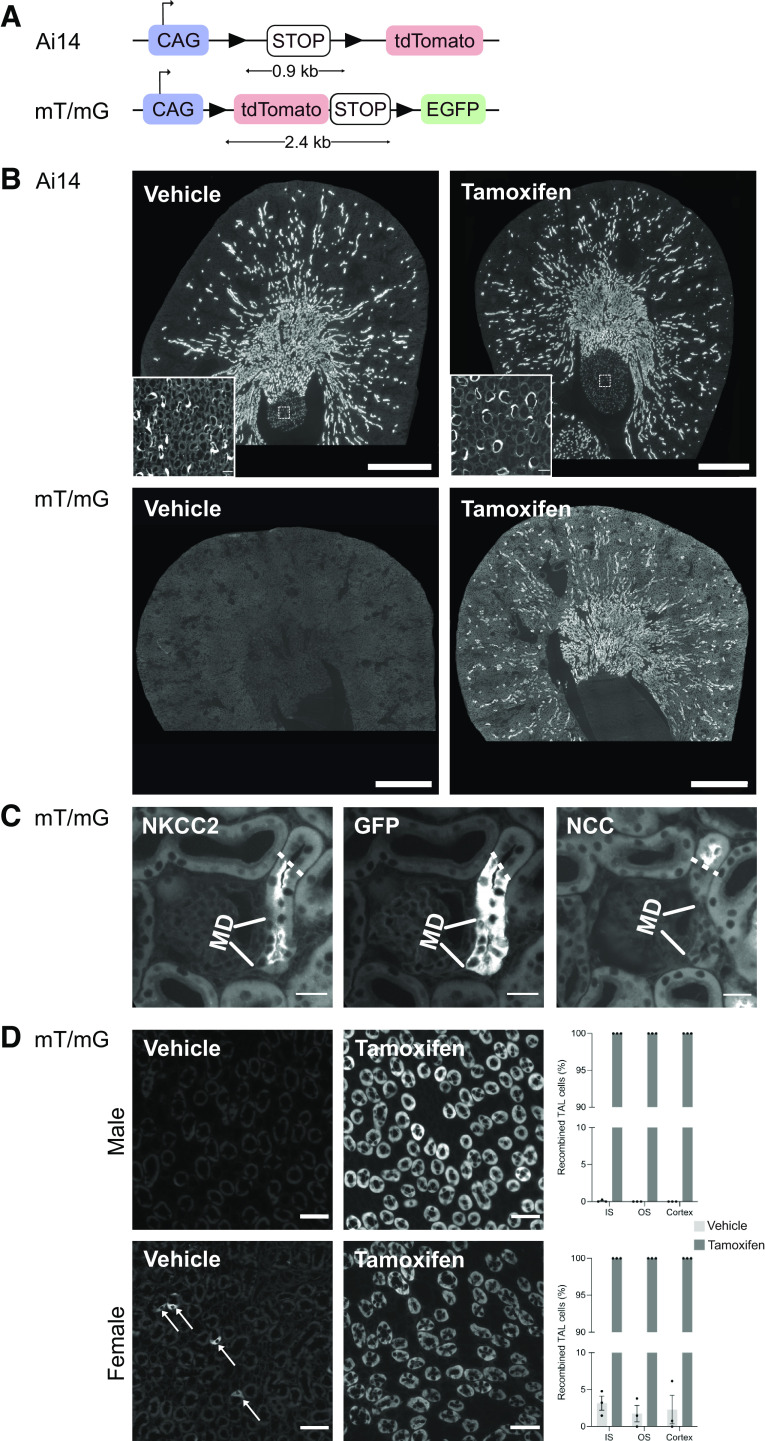
Slc12a1-CreERT-mediated DNA recombination revealed in two different reporter mouse models. *A*: reporter gene constructs in Ai14 and mT/mG mice. CAG, CAG promoter; EGFP, enhanced green fluorescent protein; STOP, stop codon. Arrowheads indicate LoxP sites. The distance (in kb) between the loxP sites is indicated. *B*: overviews of kidney cryosections from vehicle- and tamoxifen-treated Ai14^tg/+^:Slc12a1-CreERT2^tg/+^ male mice (*top*) and mT/mG^tg/tg^:Slc12a1-CreERT2^tg/+^ (*bottom*) male mice. In Ai14^tg/+^:Slc12a1-CreERT2^tg/+^ mice, strong tdTomato-related fluorescence was seen in the kidneys from both vehicle- and tamoxifen-treated mice. Thin limbs in the renal inner medulla (box and *inset*) were also fluorescent. In mT/mG^tg/tg^:Slc12a1-CreERT2^tg/+^ mice, the kidney from a vehicle-treated mouse showed no fluorescence, whereas the kidney of a tamoxifen-treated animal showed strong EGFP-related fluorescence in the renal cortex and outer medulla but not in the inner medulla. *C*: high magnifications of consecutive cryosections of the renal cortex of a tamoxifen-treated mT/mG^tg/tg^:Slc12a1-CreERT2^tg/+^ mouse. Na^+^-K^+^-2Cl^−^ cotransporter (NKCC2)-positive thick ascending limb (TAL) cells showed strong EGFP fluorescence that stopped at the transition (dashed lines) to the Na^+^-Cl^−^ cotransporter (NCC)-positive distal convoluted tubule (DCT). Also, macula densa (MD) cells showed recombination. *D*: Slc12a1-CreERT2-mediated DNA recombination in TALs of mT/mG^tg/tg^:Slc12a1-CreERT2 male (*top*) and female (*bottom*) mice after vehicle and tamoxifen administration, respectively. All immunofluorescent images show cross-sections of TALs in the inner stripe of the renal outer medulla. Vehicle-treated mice had no (male mice) or only very few (arrows in female mice) TAL cells with EGFP-related fluorescence. In contrast, tamoxifen-treated mice had strong EGFP-related fluorescence and hence recombination in all TAL cells. Quantification of fluorescent TAL cells (bar graphs) in the inner stripe (IS) and outer stripe (OS) of the outer medulla as well in the renal cortex confirmed the histological observations. Images are representative for three mice per group and were captured with a ×20 objective for the overviews in *B* and with a ×40 objective for the *insets* in *B* and for the images in *C* and *D*. Scale bars = 1,000 µm and 20 µm for the overviews and *insets* in *B*, respectively, 20 µm in *C*, and 50 µm in *D*. The percentages of TAL cells with DNA recombination (bar graphs) are expressed as means ± SE; *n* = 3 per group. When compared with a Student’s *t* test, differences between vehicle- and tamoxifen-treated mice were statistically different (*P* < 0.0001) for both sexes.

To assess the recombinase activity of Cre expression, we crossbred Slc12a1-Cre-ERT2 mice with the Ai14 reporter mouse line, which was designed to express red fluorescent tdTomato after DNA recombination (Fig. 5*A*). Surprisingly, kidneys from both tamoxifen-induced as well as vehicle-treated Slc12a1-CreERT2^wt/tg^:Ai14^wt/tg^ mice showed a bright red fluorescent signal in epithelial tubules of the renal cortex and renal outer medulla (Fig. 5*B*). Closer inspection revealed that even some thin limbs in the inner medulla harbored a fluorescent signal (Fig. 5*B*, *insets*).

Previous studies have suggested that the Ai14 reporter mouse line is particularly prone to Cre-mediated recombination, because the loxP sites are localized in close proximity (distance between loxP sites = 0.9 kb) (34). This may explain the “leakiness” of the Slc12a1-CreERT2 system that we observed in nontamoxifen-induced Slc12a1-CreERT2^wt/tg^:Ai14^wt/tg^ mice. Therefore, we used another reporter mouse line (mT/mG), in which the loxP sites are separated by a longer DNA stretch of 2.4 kb (23). This mT/mG reporter mouse line was designed to constitutively express dtTomato in all cells. After Cre-mediated recombination, red dtTomato expression is replaced by green enhanced green fluorescent protein (EGFP) expression (see gene construct in Fig. 5*A*). When we analyzed the kidneys of this Slc12a1-CreERT2^wt/tg^:mT/mG^tg/tg^ mice, the kidneys of vehicle-treated mice did not show sizeable green EGFP-related fluorescence in the renal cortex, renal outer medullar, or renal inner medulla. However, strong EGFP-related fluorescence was seen in the renal cortex and renal outer medulla of tamoxifen-induced Slc12a1-CreERT2^wt/tg^:mT/mG^tg/tg^ mice (Fig. 5*B*). Neither the inner medulla of vehicle-treated nor inner medulla of tamoxifen-treated mice was EGFP positive. Higher magnifications of the renal cortex confirmed that the EGFP signal was strictly localized to the NKCC2-positive TAL (including the macula densa) and did not extend into the NCC-positive DCT (Fig. 5*C*). Quantification of EGFP-positive cells in the kidneys of vehicle-treated Slc12a1-CreERT2^wt/tg^:mT/mG^tg/tg^ mice revealed a very low tamoxifen-independent recombination rate for both male and female mice. In male mice, tamoxifen-independent recombination was seen only in a few TAL cells (0.09 ± 0.24% TAL cells, *n* = 3) in the inner stripe of the outer medulla and was absent in the outer stripe and renal cortex (Fig. 5*D*, *top*). Female mice (Fig. 5*D*, *bottom*) had a slightly higher tamoxifen-independent recombination rate with 3.18 ± 0.78% TAL cells in the inner stripe, 1.76 ± 0.90% TAL cells in the outer stripe, and 2.31 ± 1.58% TAL cells in the cortex (*n* = 3 for each kidney zone). The tamoxifen-induced recombination rate was 100 ± 0% in TAL cells in both sexes of Slc12a1-CreERT2^wt/tg^ mice (*n* = 3 for each sex; Fig. 5*D*).

### Insertion of the CreERT2 Sequence in the Slc12a1 Gene Reduced Endogenous NKCC2 Expression

**Figure 6.**
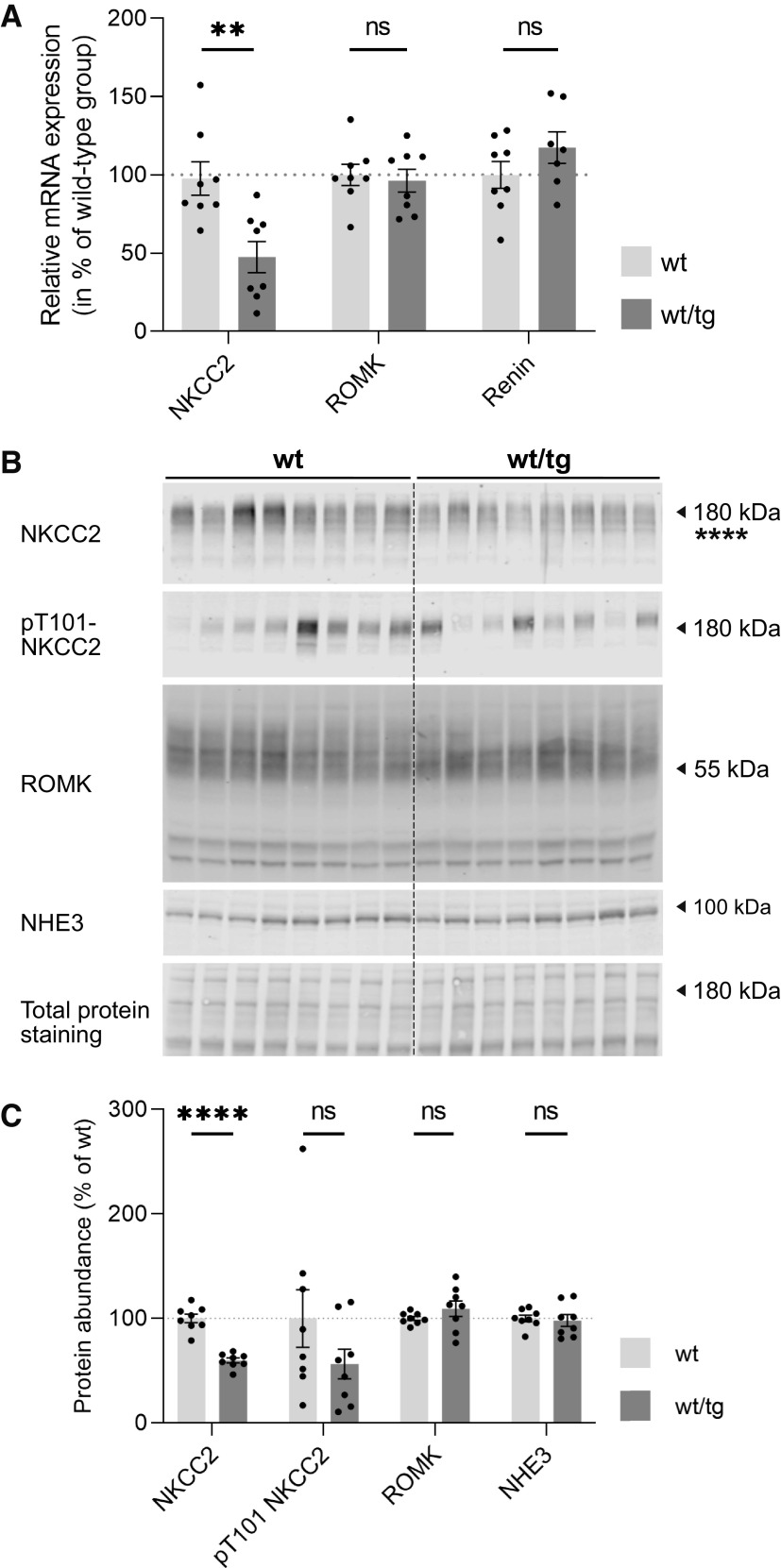
mRNA and protein expression levels for thick ascending limb (TAL) markers in total kidney homogenates of noninduced male wild-type (wt) and Slc12a1-CreERT2 mice. *A*: RT-PCR revealed that mRNA expression levels of Na^+^-K^+^-2Cl^−^ cotransporter (NKCC2), but not of renal outer medullary K^+^ (ROMK) channels and renin, were reduced in the kidneys of wild-type mice compared with Slc12a1-CreERT2^wt/tg^ mice. *B*: immunoblots for total NKCC2, phosphorylated (p)T101-NKCC2, ROMK, and Na^+^/H^+^ exchanger 3 (NHE3) in total kidney homogenates of wild-type and Slc12a1-CreERT2^wt/tg^ male mice. *C*: densitometry analysis of the immunoblots. Band signals were normalized to total protein staining with REVERT solution (see materials and methods) and expressed as a percentage of the values from wild-type mice; *n* = 8 male mice/group. Data are expressed as means ± SE and were compared using a Student’s *t* test. ****P < 0.0001; **P < 0.01. ns, not significant; tg, transgenic.

Insertion of an additional DNA sequence into the 3′-UTR may interfere with expression of the endogenous gene as the 3′-UTR impacts on mRNA localization, translation, and stability (35). Consequently, we compared NKCC2 expression levels in the kidneys of male noninduced Slc12a1-CreERT2^wt/wt^ and Slc12a1-CreERT2^tg/tg^ mice. Both NKCC2 mRNA expression (Fig. 6*A*) and NKCC2 protein abundance (Fig. 6, *B* and *C*) were lower in Slc12a1-CreERT2^wt/tg^ mice than in wild-type mice. Also, the abundance of phosphorylated NKCC2 (T101) was slightly but not significantly reduced in Slc12a1-CreERT2^wt/tg^ mice (Fig. 6, *B* and *C*). Similar observations were made in female mice (Supplemental Fig. S1). In contrast to NKCC2, expression of other relevant TAL gene products, such as the ROMK channel, the Na^+^/H^+^ exchanger 3, the Cl^−^ channel ClC-Kb, the Ca^2+^-sensing receptor, and the secreted protein uromodulin (36) did not differ significantly between kidneys from male wild-type and Slc12a1-CreERT2^wt/tg^ mice (Fig. 6 and Supplemental Table S2), suggesting a specific effect of transgene expression on the NKCC2 locus.

### Lower Abundance of NKCC2 in Slc12a1-Cre-ERT2 Mice Had No Major Impact on TAL Function

**Figure 7.**
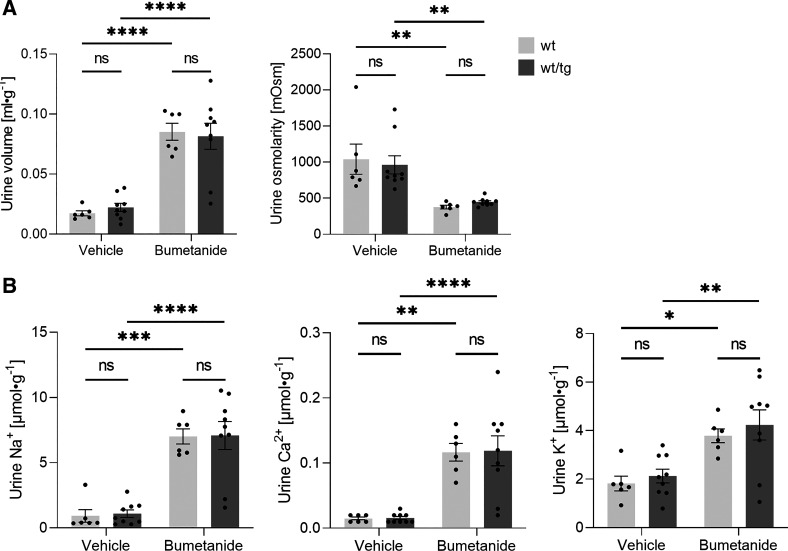
Urinary volume, osmolarity, and ion excretion in vehicle- and bumetanide-treated noninduced wild-type (wt) and Slc12a1-CreERT2^wt/tg^ male mice. *A*: urine volume increased and urine osmolarity decreased in response to a single intraperitoneal injection of bumetanide in both wild-type and Slc12a1-CreERT2^wt/tg^ mice. *B*: urinary Na^+^, Ca^2+^, and K^+^ excretion increased in response to a single intraperitoneal injection of bumetanide in both wild-type and Slc12a1-CreERT2^wt/tg^ mice. Urine was collected after the injections for 4 h. *n* = 6 for wild-type mice and *n* = 9 for Slc12a1-CreERT2^wt/tg^ mice. Data are expressed as means ± SE and were compared using a two-way ANOVA test. ****P < 0.0001; ***P < 0.001; **P < 0.01; *P < 0.05. ns, not significant; tg, transgenic.

As outlined in introduction, NKCC2 is critical for whole body water and ion balance. Hence, reduced NKCC2 expression may cause renal water and salt wasting with impaired homeostasis. Therefore, we compared the renal function of noninduced Slc12a1-CreERT2^wt/wt^ mice (*n* = 6 male mice) with one of the groups of noninduced Slc12a1-CreERT2^wt/tg^ Slc12a1-CreERT2 mice (*n* = 9 male mice) by assessing water and food intake, 24-h urine volume, urine osmolality, urine ion excretion, blood ion concentrations, and blood acid-base status. As shown in Table 2, the two groups of mice did not significantly differ from each other for any parameter. Consistent with a maintained salt balance, transgenic mice had the same renal renin expression, plasma aldosterone concentration, and urinary aldosterone excretion as wild-type mice (Fig. 6*A* and Table 2). Even when challenged by mild water restriction for 24 h, heterozygous Slc12a1-CreERT2 mice were able to concentrate the urine as efficient as wild-type mice, and body weights remained similar for both groups (Table 3). To further assess TAL function, we also performed a bumetanide test as described in materials and methods. The single bumetanide injection caused a profound increase of urine flow and urinary Na^+^, K^+^, and Ca^2+^ excretion as well as a drop in urine osmolarity that were similar for both groups of mice (Fig. 7), further suggesting that the lowered abundance of NKCC2 in Slc12a1-Cre-ERT2 mice had no major impact on TAL function.

**Table 3.** Body weight, food/water intake, and urine parameters under mild water restriction

	wt (*n* = 6)	wt/tg (*n* = 9)	*P* Value
Body weight, g	27.7 ± 0.2	26.9 ± 0.3	0.2119
Water intake, mL·g body wt^−1^	0.13 ± 0.00	0.13 ± 0.01	0.9704
Food intake, g·g body wt^−1^	0.13 ± 0.02	0.13 ± 0.03	0.9055
Urine volume, mL·g body wt^−1^	0.06 ± 0.01	0.07 ± 0.00	0.3515
Urine osmolality, mosmol/kgH_2_O	2852 ± 183	2497 ± 121	0.4278
Urine creatinine, μmol·g body wt^−1^	0.16 ± 0.00	0.20 ± 0.00	0.0018
Urine Na^+^, μmol·g body wt^−1^	8.48 ± 0.35	9.97 ± 0.47	0.1803
Urine K^+^, μmol·g body wt^−1^	19.75 ± 0.97	23.19 ± 1.07	0.2216
Urine Ca^2+^, μmol·g body wt^−1^	0.09 ± 0.01	0.13 ± 0.01	0.1082
Urine Mg^2+^, μmol·g body wt^−1^	1.64 ± 0.15	1.84 ± 0.13	0.5973

Data are from male mice and are expressed as means ± SE. Food and water intake and creatinine were normalized to body weight. Urine ions and creatinine were normalized to body weight and to urine volume. Urine was collected over 24 h. Data were compared using a Student’s *t* test. tg, transgenic; wt, wild-type.

### Lower Abundance of NKCC2 in Slc12a1-Cre-ERT2 Mice Had No or Only Limited Impact on Ion Transporters/Channels Downstream of the TAL

**Figure 8.**
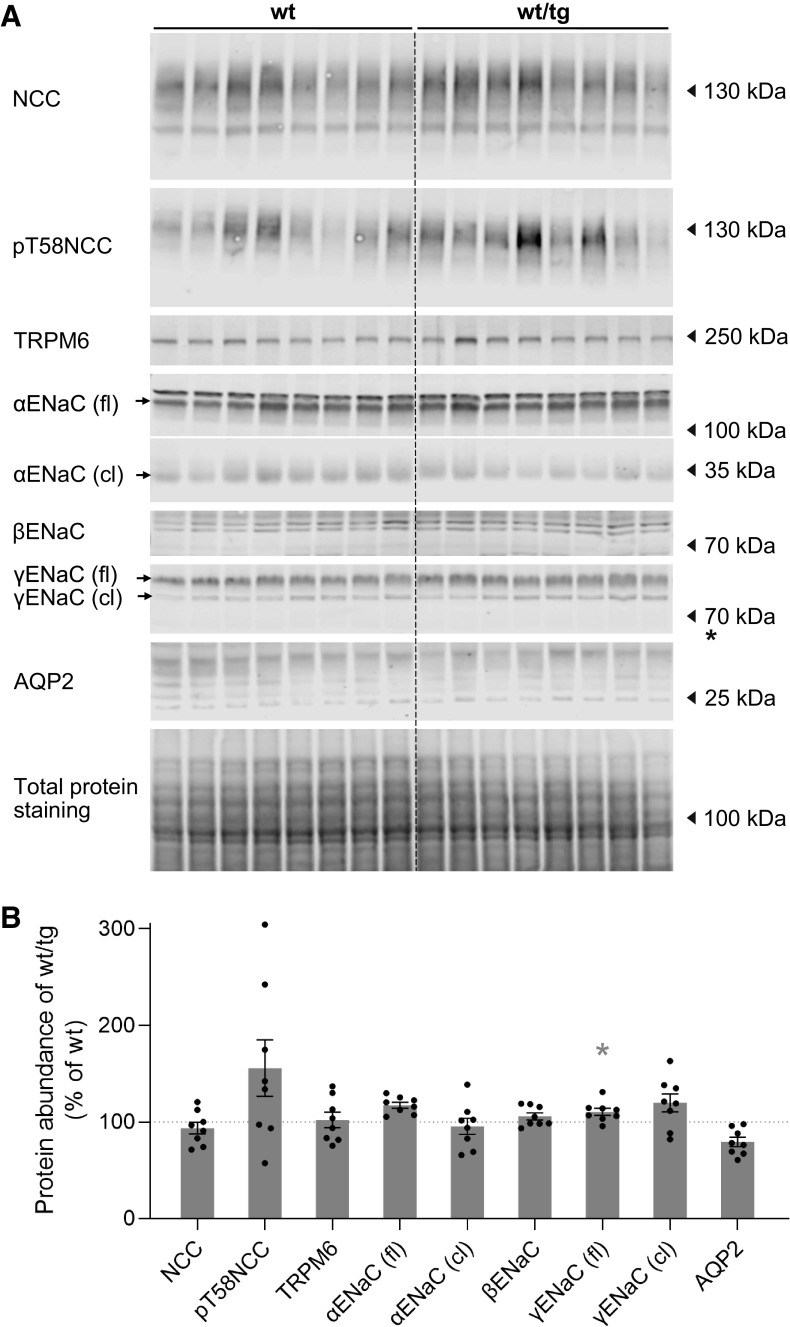
Protein expression levels of ion transporters and ion and water channels expressed in renal tubules downstream of the thick ascending limb. *A*: immunoblots for Na^+^-Cl^−^ cotransporter [NCC; total + phosphorylated (p-)T58], transient receptor potential cation channel subfamily M member 6 (TRPM6), epithelial Na^+^ channel (ENaC; α-, β-, and γ-subunits), and aquaporin-2 (AQP2) in total kidney homogenates of noninduced wild-type (wt) and Slc12a1-CreERT2^wt/tg^ male mice. *B*: densitometry analysis of the immunoblots. Band signals were normalized to total protein staining with REVERT solution (see materials and methods) and expressed as a percentage of the values from wild-type mice; *n* = 8 male mice/group. Data are expressed as means ± SE and were compared using a Student’s *t* test. *P < 0.05. tg, transgenic.

Previous studies have demonstrated that impaired TAL function might be compensated, at least in part, by an upregulation of ion transport pathways in the downstream localized nephron segments (28, 37, 38). Therefore, we assessed the abundance of total NCC, phosphorylated NCC (pT58), α-, β-, and γ-subunits of ENaC, Mg^2+^ channel transient receptor potential cation channel subfamily M member 6, and water channel aquaporin-2 (Aqp2; Fig. 8 and Supplemental Table S2). Except for a slight but statistically not significant upregulation of NCC phosphorylation and a slight upregulation of total γ-ENaC at the protein level, no consistent differences were found between wild-type and transgenic mice.

## DISCUSSION

This study describes and characterizes a new gene-modified mouse model for inducible and highly efficient gene targeting in the TAL. In this mouse line, tamoxifen-sensitive CreERT2 is inserted into the 3′-UTR of the endogenous Slc12a1 (NKCC2) locus. In contrast to previously developed mouse lines, in which Cre recombinase was expressed under the control of constitutively active uromodulin (18, 19) or Slc12a1 (NKCC2) (21) promotors, Slc12a1-CreERT2^wt/tg^ mice allow for both good spatial and also very efficient temporal control of gene deletion.

Adult Slc12a1-CreERT2^wt/tg^ mice have strong Cre expression along the entire TAL. Cre expression starts precisely at the transition from the thin ascending limb to the TAL and stops at the border to the DCT. Cre expression is also strong in the macula densa and thus also allows targeting of this critical signaling spot for tubuloglomerular feedback (2). Consistent with the high levels of Cre expression along the entire TAL, the Slc12a1-CreERT2^wt/tg^ mouse model achieves complete (∼100%) gene recombination when crossed with the appropriate reporter mouse line. This very efficient gene targeting is likely related to the very strong expression of Slc12a1 (NKCC2) in the TAL. In fact, RNA sequencing of microdissected nephron segments revealed that Slc12a1 is one of the most abundant gene products (top 25) in the mouse TAL with mean transcript per million expression values of 4116.3 and 6260.9 for the medullary and cortical TAL, respectively (39).

A major concern with the use of inducible CreERT2 mouse lines is unwanted Cre-mediated recombination that occurs already without tamoxifen application (40). Also in our study, we observed a very high leakiness when Slc12a1-CreERT2^wt/tg^ mice were crossed with the Ai14 reporter mouse line. However, when Slc12a1-CreERT2^wt/tg^ mice were crossed with the mT/mG reporter line, almost no recombination was observed as long no tamoxifen was applied. This sharp difference between the two reporter mouse lines is consistent with previous findings (16, 34, 41) that suggested that the distance between the two loxP sites in the genome determines the recombination threshold. The longer this distance is, the better is the “tightness” of the gene-targeting system (34, 41). In fact, the distance between the two loxP sites is much shorter in Ai14 reporter mice (0.9 kb) than in mT/mG mice (2.4 kb). Interestingly, not only the distance between the two loxP sites but also the sex of the mice impacted the background recombination rate in our mouse model. Although noninduced Slc12a1-CreERT2^wt/tg^:mT/mG male mice had no tamoxifen-independent recombination, their female counterparts revealed a background recombination rate of ∼3% in TAL cells. Although ERT has an ∼1,000-fold lower estrogen-binding activity than the ER (42), it is tempting to speculate that the high endogenous estrogen level in female mice explains the observed sex difference. Unfortunately, possible sex differences in tamoxifen-independent Cre-ERT2-mediated recombination are not routinely assessed in gene-targeting studies. However, two recent studies did not observe significant differences in tamoxifen-independent recombination rates between male and female CreERT2 mice (16, 34).

The high leakiness observed in Ai14^wt/tg^ Slc12a1-CreERT2^wt/tg^ mice may also explain why these mice also show recombination in the thin ascending limbs. In adult mice, NKCC2 is not expressed in the thin limbs and hence cannot explain the targeting of this nephron portion (43). However, during renal development, ascending thin limb cells derive via transdifferentiation from NKCC2-positive progenitor cells in the end portion of the developing TAL (44). Tamoxifen-independent Cre-mediated in these progenitor cells likely transmits the gene modification to all progeny including the forming thin limbs.

A weakness of Slc12a1-CreERT2^wt/tg^ mice is the reduced NKCC2 mRNA and protein expression compared with wild-type mice. Consistent with observations in heterozygous NKCC2 knockout animals (NKCC2^+/−^) (45), the slight reduction of NKCC2 abundance, however, has no major effect on overall renal function, as indicated by the unchanged urinary ion excretions, plasma aldosterone levels, renal renin expression, urinary concentration under baseline conditions and water restriction, and normal bumetanide response. Nevertheless, the slight upregulation of NCC and ENaC in heterozygous Slc12a1-CreERT2^wt/tg^ mice suggests some impact on TAL function that is compensated in nephron portions downstream to the TAL (i.e., the DCT, connecting tubule, and collecting duct), consistent with similar observations in loop diuretic-treated animals (37, 38). The precise molecular mechanism for the downregulation of NKCC2 at the mRNA and protein levels is unclear, but it is likely related to the insertion of Cre recombinase into the 3′-UTR. The 3′-UTR is known to impact on mRNA transcription, localization, and stability as well as on protein translation and posttranslational modifications (35, 46). However, we cannot exclude that the reduced NKCC2 expression is a direct negative effect of Cre expression on TAL cells. Previous studies have reported that Cre recombinase may even induce DNA damage in the absence of LoxP sites (47). However, this effect would likely affect not only expression of NKCC2 but also expression of other TAL proteins including uromodulin, Ca^2+^-sensing receptor, ClC-Kb, and ROMK, which we did not observe. Interestingly, Chen et al. (48) reported a reduction of endogenous Aqp2 expression in their Aqp2-creERT2^wt/tg^ mice, whereas we did not see altered NCC (Slc12a3) expression in our previously reported Slc12a3-CreERT2^wt/tg^ mouse line (15).

Another notable peculiarity of the Slc12a1-CreERT2^wt/tg^ mouse model is the abundance of Cre recombinase in the testes. As confirmed in our experiments, NKCC2 is absent in the testes and hence cannot drive Cre expression at this site. Interestingly, unintended Cre expression in the testis has also been reported for mice in which Cre recombinase is expressed under the uromodulin promoter (19). Likewise, our recently described Slc12a3-CreERT2 (NCC-Cre) mice (15) show Cre expression in the testis (Supplemental Fig. S2). The underlying mechanism for this ectopic Cre expression in the testis is unclear but needs to be considered in the experimental design as it may go along with gene deletion in the testis and/or impact on androgen production as indicated by the findings of the present study (Table 2).

Because of the slight reduction of NKCC2 expression in the kidney and ectopic Cre expression in the testis, we recommend that in gene-targeting studies the Slc12a1-CreERT2^wt/tg^ allele is always included in both the control and knockout groups. Of course, this requires that additional experiments control for possible confounding effects of the insertion of loxP sites into the genome (49) and/or for off-target effects of tamoxifen (50). As a selective ER modulator, tamoxifen and its active metabolites may impact on the function of several organs including the bone, heart, liver, and gastrointestinal tract (50). Likewise, several renal ion channels and transport proteins are potential targets for the regulation by ovarian hormones and tamoxifen (32, 33, 51–53). In our study, we observed only mild effects on TAL-specific NKCC2 and DCT-specific NCC, although we analyzed the kidneys immediately after the last tamoxifen application. Nevertheless, even with longer latency periods, unintended effects of tamoxifen on ion transporters and other physiological parameters need to be considered, in particular as tamoxifen accumulates in the body after repeated application (54). The long washout of tamoxifen may also explain why we observed a rather slow redistribution of Cre recombinase from the cell nuclei to the cytoplasm of TAL cells after completion of 5 days of repeated tamoxifen gavage.

In summary, we describe here a novel gene-modified mouse model (Slc12a1-CreERT2^wt/tg^) for highly specific, efficient, and inducible gene targeting in the TAL that promises to be a valuable experimental tool for the analysis of the role of candidate genes and signaling cascades controlling TAL function.

## DATA AVAILABILITY

Data will be made available upon reasonable request.

## SUPPLEMENTAL DATA

10.6084/m9.figshare.22127720.v1 Supplemental Figs. S1 and S2 and Supplemental Tables S1 and S2: https://doi.org/10.6084/m9.figshare.22127720.v1.

## GRANTS

The research of J.L. is supported by the Swiss National Science Foundation 310030_143929/1, the Swiss National Centre for Competence in Research “Kidney.CH”, and the clinical research priority program HYRENE of the medical faculty of the University of Zurich.

## DISCLOSURES

No conflicts of interest, financial or otherwise, are declared by the authors.

## AUTHOR CONTRIBUTIONS

D.L.-C. and J.L. conceived and designed research; L.B. and D.V.W. performed experiments; L.B., A.O., D.L.-C., and J.L. analyzed data; L.B., A.O., D.L.-C., and J.L. interpreted results of experiments; L.B. prepared figures; L.B. drafted manuscript; L.B., D.V.W., A.O., D.L.-C., and J.L. edited and revised manuscript; L.B., D.V.W., A.O., D.L.-C., and J.L. approved final version of manuscript.
